# Dual-targeting nanozyme for tumor activatable photo-chemodynamic theranostics

**DOI:** 10.1186/s12951-022-01662-9

**Published:** 2022-11-03

**Authors:** Chaoyi Chen, Yuwen Chen, Lulu Zhang, Xuanhao Wang, Qingshuang Tang, Yan Luo, Yuan Wang, Cheng Ma, Xiaolong Liang

**Affiliations:** 1 Department of Ultrasound, Peking University Third Hospital, 49 North Garden Rd., Haidian District Beijing, 100191 Beijing, China; 2 Department of Electronic Engineering, Beijing National Research Center for Information Science and Technology, Tsinghua University, 100084 Beijing, China; 3 Institute for Precision Healthcare, Tsinghua University, 100084 Beijing, China

**Keywords:** Nanozyme, Fenton reaction, Photoacoustic imaging, Photothermal therapy, Chemodynamic therapy

## Abstract

**Abstract:**

Tumor phototheranostics holds a great promise on account of its high spatiotemporal resolution, tumor-specificity, and noninvasiveness. However, physical limitation of light penetration and “always on” properties of conventional photothermal-conversion agents usually cause difficulty in accurate diagnosis and completely elimination of tumor. Meanwhile, nanozymes mediated Fenton reactions can well utilize the tumor microenvironment (TME) to generate hydroxyl radicals for chemodynamic therapy (CDT), but limited by the concentration of H_2_O_2_ in TME and the delivery efficiency of nanozymes. To overcome these problems, a dual-targeting nanozyme (FTRNPs) is developed for tumor-specific in situ theranostics, based upon the assembling of ultrasmall Fe_3_O_4_ nanoparticles, 3,3’,5,5’-tetrameth-ylbenzidine (TMB) and the RGD peptide. The FTRNPs after H_2_O_2_ treatment exhibits superior photothermal stability and high photothermal conversion efficiency (η = 50.9%). FTRNPs shows extraordinary accumulation and retention in the tumor site by biological/physical dual-targeting, which is 3.54-fold higher than that without active targeting. Cascade-dual-response to TME for nanozymes mediated Fenton reactions and TMB oxidation further improves the accuracy of both photoacoustic imaging and photothermal therapy (PTT). The tumor inhibition rate of photo-chemodynamic therapy is ~ 97.76%, which is ~ 4-fold higher than that of PTT or CDT only. Thus, the combination of CDT and PTT to construct “turn on” nanoplatform is of great significance to overcome their respective limitations. Considering its optimized “all-in-one” performance, this new nanoplatform is expected to provide an advanced theranostic strategy for the future treatment of cancers.

**Graphical abstract:**

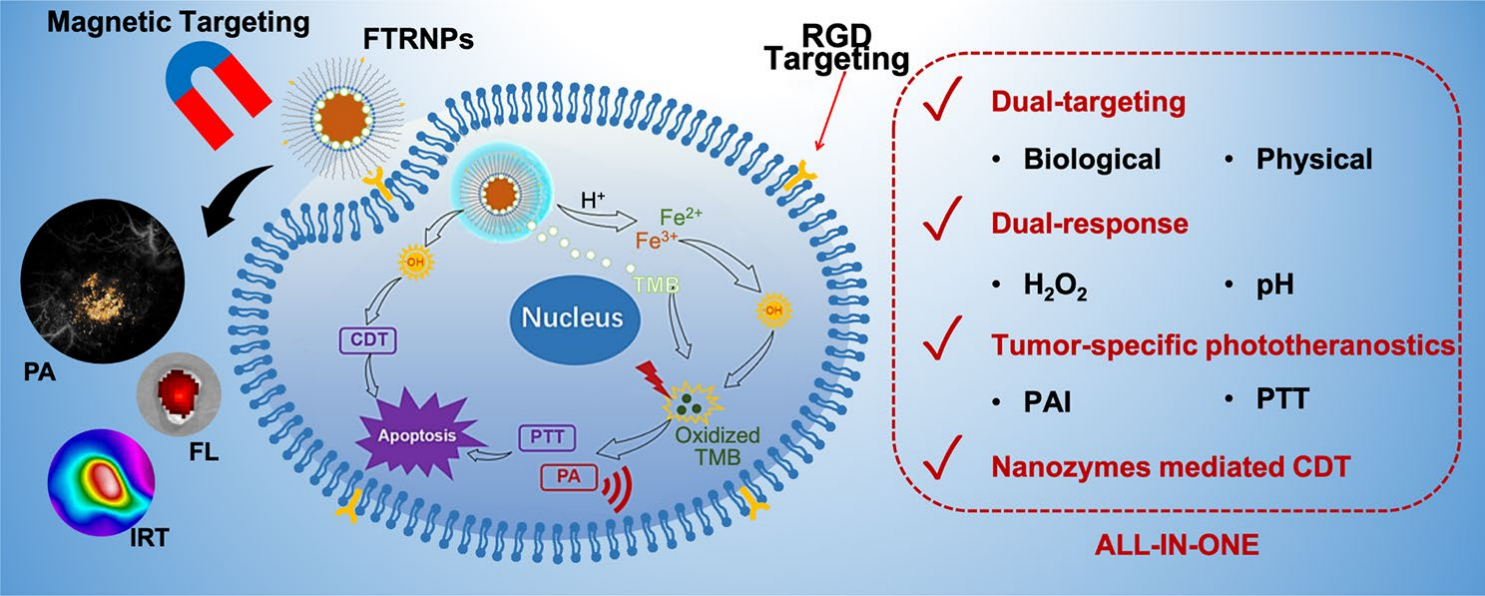

**Supplementary Information:**

The online version contains supplementary material available at 10.1186/s12951-022-01662-9.

## Introduction

Cancer is a main threat to human life and an obstacle to extending life expectancy [1, 2]. Therefore, scientists have been committed to exploring effective diagnostic and treatment strategies to overcome the threat of cancer. Among the image-based diagnosis technologies, photoacoustic (PA) imaging (PAI) has the advantages of high spatial resolution, rich contrast, and biosafety [3–5]. PAI combined with exogenous contrast agents has been widely used in cancer diagnosis [6]. Exogenous PA contrast agents, such as near-infrared (NIR) dyes [7–10], carbon nanomaterials [11, 12], semiconducting polymer nanoparticles (NPs) [13, 14] and metallic NPs [15, 16], can provide outstanding PA contrasts by the conversion of absorbed light energy into PA signals. A related technique, photothermal therapy (PTT), converts the absorbed light into hyperthermia through photothermal-conversion NPs, which can ablate tumor tissue under NIR irradiation [17, 18]. Due to the similarity between PA agents and PTT nanomaterials, PAI-guided PTT has been widely explored, so as to realize the theranostic of cancers. However, those photothermal-conversion NPs cannot achieve accurate theranostics due to their “always on” properties. The unique characteristics of tumor microenvironment (TME), such as high redox state, mild acidity and hypoxia, allow PAI/PTT agents to be selectively “turned on”, with the benefit of reduced false-positive image signals and treatment side effects [15, 19–23]. As a standard and safe chromogenic compound widely used in the clinical research, 3,3’,5,5’-tetramethylbenzidine (TMB) enjoys the “turn on” property for tumor-specific PAI-guided PTT [24, 25]. Oxidized TMB has a strong NIR absorption and can respond sensitively to the pH change of the environment. Recently, PAI-guided PTT was achieved by redox activation and acid enhanced agents prepared based on horseradish peroxidase and TMB [26]. Furthermore, in order to avoid the relatively poor stability of natural horseradish peroxidase in the presence of certain inhibitors and elevated temperature, a nanoreactor using PtAu nanozyme instead of natural horseradish peroxidase was designed [27]. However, such nanoreactors exhibited relatively low chromogenic efficiency. At the same time, formulations using heavy metals may limit further clinical application due to low solubility and toxicity concerns [28, 29].

Fe_3_O_4_ NPs possessing superparamagnetic properties at certain sizes have been successfully used for imaging, drug delivery and thermal therapy, and has been used as an inorganic nanomedicine approved by the U.S. Food and Drug Administration (FDA) [29, 30]. H_2_O_2_, the key redox parameter of TME, can be decomposed by nanozymes to produce hydroxyl radicals (•OH) in weak acidic TME. Some nanozymes with outstanding catalytic efficiency for tumor CDT and tumor hypoxia relief have been reported [31–33]. Fe_3_O_4_ NPs are also involved in peroxidase-like activity, which can produce •OH via Fenton reactions. Therefore, Fe_3_O_4_ NPs have the potential to oxidize TMB in situ in the tumor, similar to natural horseradish peroxidase and heavy metal nanozymes [34, 35]. In recent years, using •OH produced by Fenton/Fenton-like reaction to trigger tumor cell apoptosis and inhibit tumor growth in situ is a newly developed treatment strategy, called chemodynamic therapy (CDT) [36, 37]. Therefore, the therapeutic effect of CDT depends on the rate of Fenton reactions, which depends not only on the concentration of H_2_O_2_, but also on the performance of the Fenton catalyst. To date, the low H_2_O_2_ concentration and the poor catalytic efficiency of Fenton catalyst available in the TME are the main limiting factors preventing CDT from successful clinical translations [35, 38].

An extra benefit provided by the Fe_3_O_4_-based NPs is their superparamagnetism which enables active targeting through the use of an external magnetic field. Magnetic targeting can not only increase the accumulation of the particles inside tumors, but also facilitate their uptake by cells [39, 40]. Besides, modifying tumor cell targeting moieties on the NP surface is a widely recognized method to improve the specificity and effectiveness of the NP uptake [41]. For example, the RGD (Arg-Gly-Asp) peptide has excellent active targeting ability due to its specific binding to αvβ3 integrin, which is highly expressed on tumor cells and tumor neovascular endothelial cells [39, 41–43]. Therefore, developing “all-in-one” nanoplatform with tumor-specificity and high-targeting ability for CDT enhanced phototheranostics is of great significance to improve the theranostic efficiency.

**Fig. 1:**
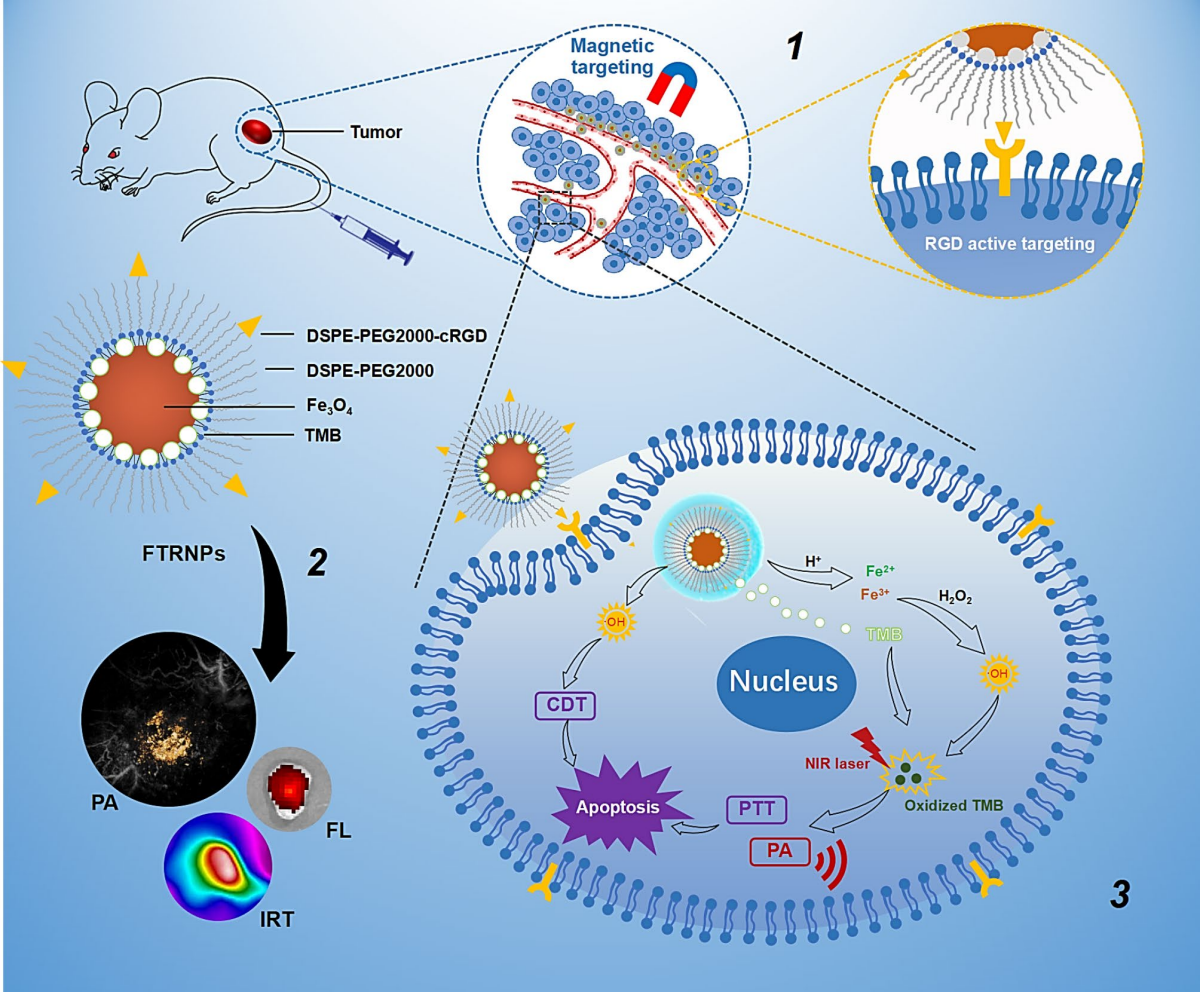
Schematic representation of tumor-specific photo-chemodynamic theranostics via dual-targeting nanozyme. (1) Dual-targeting process of FTRNPs to the tumor site. (2) Dual-responsive in situ tumor-specific imaging: PAI, FL imaging and IR thermal imaging. (3) Synergistic therapy of CDT based on Fe_3_O_4_ mediated Fenton reactions and PTT based on oxidized TMB.

Herein, we constructed a dual-targeting nanozyme (Fe_3_O_4_/TMB-PEG-RGD, FTRNPs) by assembling Fe_3_O_4_ NPs, TMB, and RGD. FTRNPs could provide tumor-specific PTT in situ, guided by molecular PAI and enhanced by nanozyme-mediated CDT. High specificity and effectiveness were enabled by both active biological and physical dual-targeting strategy (Fig. 1). The combination of Fe_3_O_4_ nanozyme and TMB allowed FTRNPs to be “switched on” inside tumor, through a cascade of TME responsive processes triggered by the overexpressed H_2_O_2_ and acidic level in tumor. The chromogenic ability of FTRNPs in situ at the tumor promised to improve the specificity of PAI. In addition, the switchable property of the FTRNPs to be “turned on” could well improve the accuracy of PTT as compared to the conventional PTT using “always on” photothermal agents. Meanwhile, the combined use of biological active targeting (RGD) and physical active targeting (magnetic targeting) substantially improved the accumulation and retention of FTRNPs at the tumor site, as proved by comparing FTRNPs with Fe_3_O_4_/TMB-PEG (FTNPs) through three-dimensional PA mesoscopic imaging (PAMe). More importantly, the excellent targeting performance and the presence of nanozyme-mediated CDT made up for the limitation of PTT. Considering its optimized “all-in-one” performance, this new nanoplatform was expected to provide an advanced theranostic strategy for the future treatment of cancers.

## Results and discussion

### Fabrication and characterizations

**Fig. 2:**
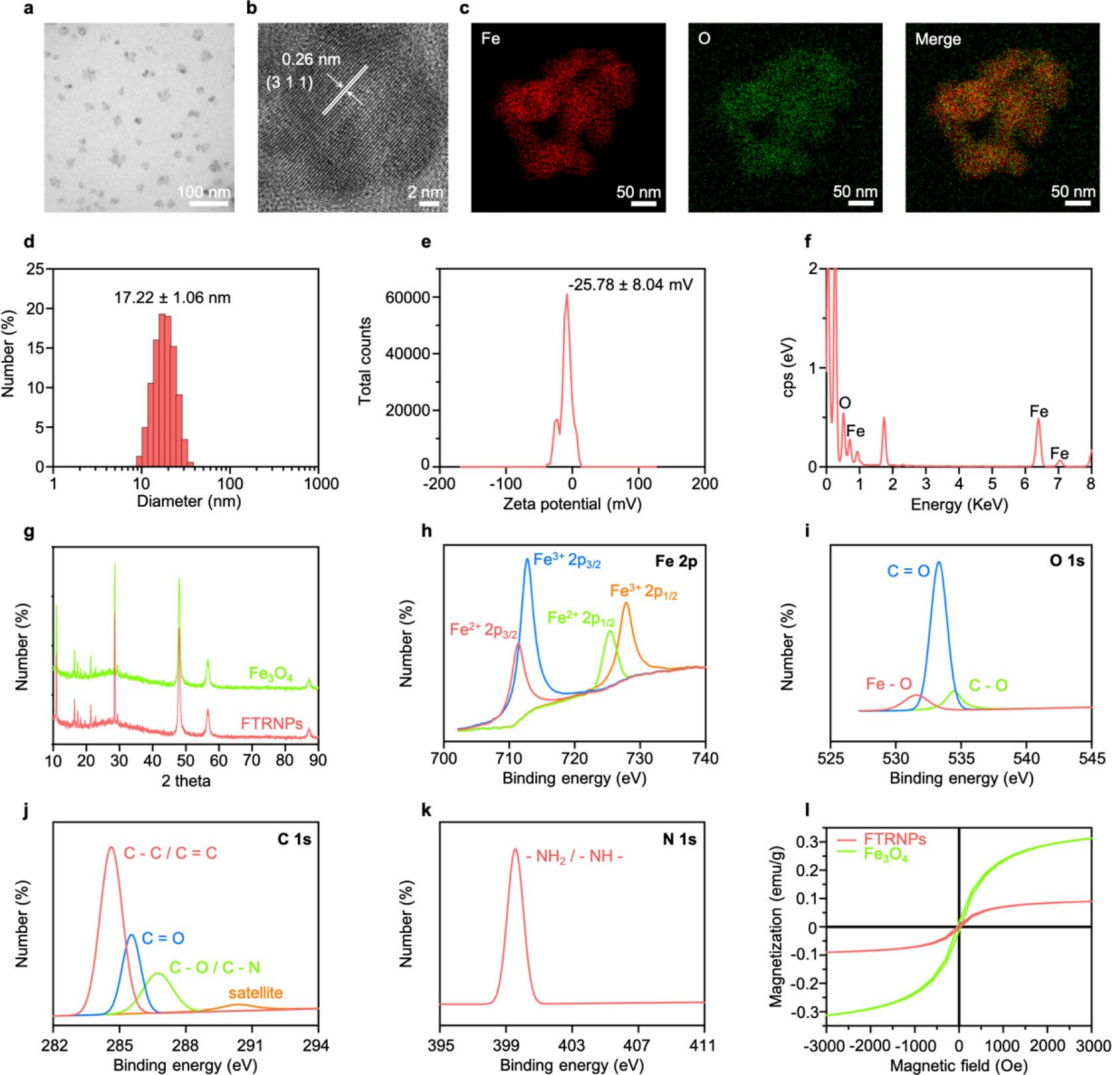
Characterization of FTRNPs. (a) TEM image of FTRNPs. (b) High resolution TEM image of FTRNPs. (c) Elemental mapping of Fe, O and FTRNPs. (d) Hydrodynamic size distribution of FTRNPs. (e) Zeta potential of FTRNPs. (f) EDS spectrum of FTRNPs. (g) XRD patterns of hydrophobic Fe_3_O_4_ and FTRNPs. (h-k) XPS spectra of Fe 2p, O 1s, C 1s and N 1s. (l) Comparison of the magnetization response curves of hydrophobic Fe_3_O_4_ and FTRNPs.

Both FTNPs and FTRNPs were prepared through a thin-film rehydration method [44, 45]. FTNPs were prepared by assembly of DSPE-PEG2000 with hydrophobic Fe_3_O_4_ and TMB (1.8:1:0.1, w/w), resulting in NPs with good water dispersibility and TMB loading efficiency of 97.6%. For FTRNPs, a small amount of DSPE-PEG2000-cRGD (~ 10% of phospholipid) was added for biological active targeting during the preparation. Transmission electron microscope (TEM) results showed that FTRNPs exhibited uniform distribution with an average size of 16.41 ± 3.19 nm (Fig. 2a), while hydrophobic Fe_3_O_4_ showed an average size of 6.99 ± 1.16 nm (Fig. S1). High resolution TEM showed the crystallinity of FTRNPs’ lattice parameter was ~ 0.26 nm, which accorded with the (3 1 1) plane of Fe_3_O_4_ nanocrystals (Fig. 2b). The area elemental mapping of FTRNPs confirmed the uniform distribution of Fe and O (Fig. 2c). The hydrodynamic size and the zeta potential of FTRNPs in aqueous solutions were 17.22 ± 1.06 nm and − 25.78 ± 8.04 mV, respectively, as determined by the dynamic light scattering (DLS) measurement (Fig. 2d, e). The composition of the FTRNPs was confirmed by the energy dispersive X-ray spectrum (EDS) (Fig. 2f). X-ray diffraction (XRD) peaks of hydrophobic Fe_3_O_4_ and FTRNPs indicated the consistency before and after fabrication (Fig. 2 g). X-ray photoelectron spectroscopy (XPS) analysis of FTRNPs confirmed its integration of Fe_3_O_4_, TMB and phospholipid (Fig. 2 h-k). Meanwhile, FTRNPs exhibited excellent magnetic performance. In the presence of magnetic field, the sample could respond quickly and moved to the magnet side (Fig. S2). The superparamagnetism of FTRNPs measured by vibrating sample magnetometry showed that they remained magnetized after the assembly, which made it possible for magnetic targeting (Fig. 2 L).

### H_2_O_2_ responsiveness and sensitivity of FTRNPs at acidic condition

**Fig. 3:**
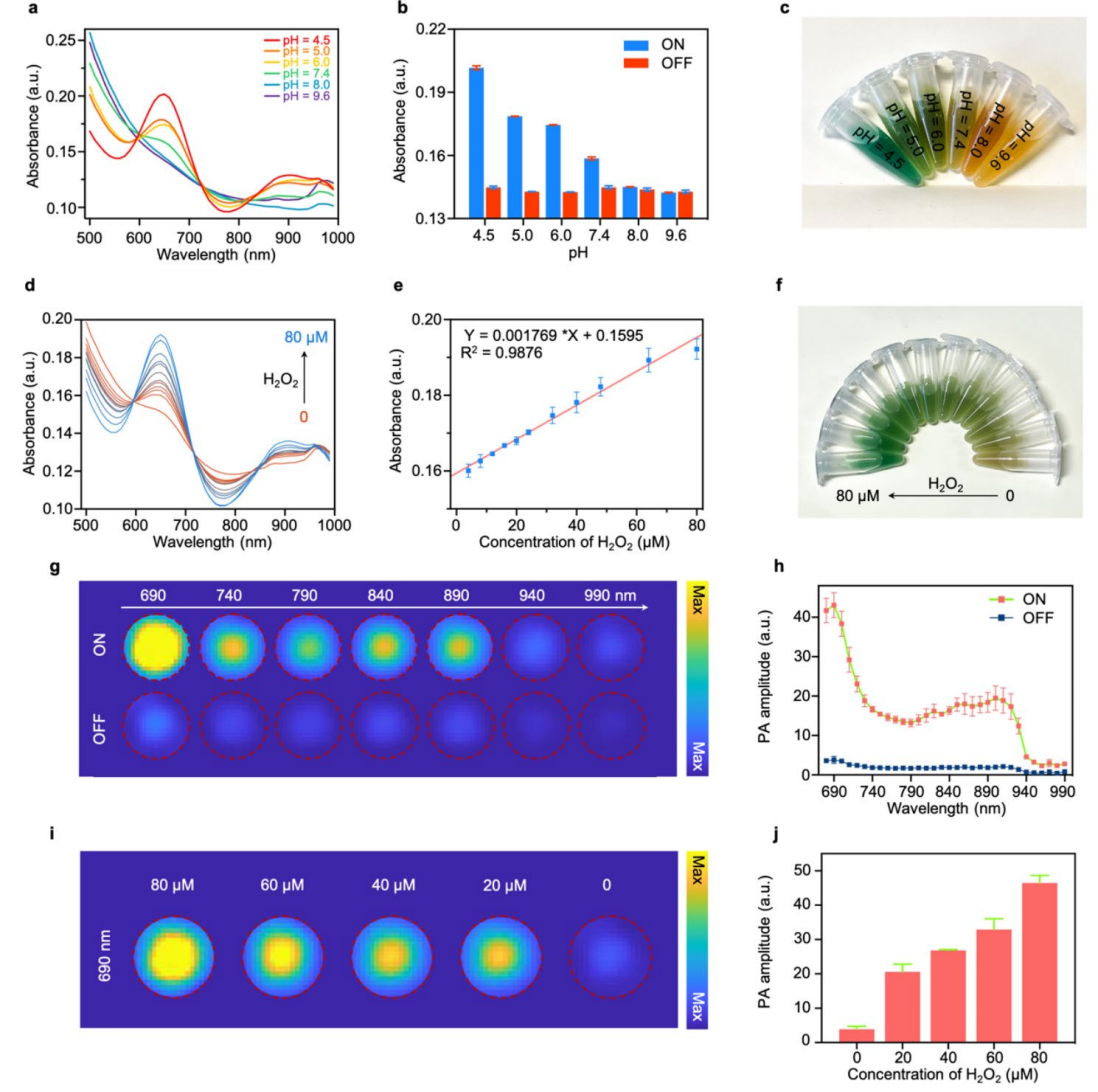
Responsiveness and sensitivity of FTRNPs. (a) UV-vis absorption spectra of H_2_O_2_-treated FTRNPs at different pH levels. (b) The absorbance of FTRNPs at 650 nm at different pH levels before (off) and after “turning on” by H_2_O_2_.(off: without H_2_O_2_-treatment, on: with H_2_O_2_-treatment) (c) Photograph of FTRNPs solution at different pH levels after “turning on” by H_2_O_2_. (d) UV-vis absorption spectra of FTRNPs upon addition of H_2_O_2_ at different concentrations. (e) The absorbance of FTRNPs at 650 nm as a function of H_2_O_2_ concentrations. (f) Photograph of FTRNPs solution after “turn on” by H_2_O_2_ (0–80 µM). (g) PA images of FTRNPs before and after “turning on” by H_2_O_2_. (h) PA spectra of FTRNPs before and after “turning on” by H_2_O_2_. (i) PA images of FTRNPs after “turning on” by H_2_O_2_ (0–80 µM). (j) The PA amplitude of FTRNPs at 690 nm after “turning on” by H_2_O_2_ (0–80 µM)

In the mild acidic TME, Fe_3_O_4_ NPs can decompose H_2_O_2_ to generate •OH through Fenton reactions [31, 33]. Subsequently, the colorless TMB in FTRNPs could be oxidized by those •OH to “turn on” the FTRNPs by forming oxidized TMB with strong absorption in NIR region. The spectral switching of FTRNPs made tumor-specific PAI and PTT possible. Both Fe_3_O_4_ mediated Fenton reaction and TMB oxidation required acidic conditions. The absorbances of FTRNPs in different pH buffer solutions containing H_2_O_2_ (80 µM) were determined (Fig. 3a), and the absorbance increase at 650 nm was found to be pH-dependent (Fig. 3b). Moreover, the color of the solution deepened with the increase of acidity (Fig. 3c).

To investigate the H_2_O_2_ responsiveness of FTRNPs, H_2_O_2_ of different concentrations (0 − 80 µM) was added into FTRNPs solutions under mild acidic conditions (pH = 6.0). The spectra of the mixed solutions exhibited broad absorption in NIR spectral range with two absorptions peaked at ~ 650 nm and ~ 900 nm, and the absorbance increased with the concentrations of H_2_O_2_ (Fig. 3d). Moreover, the absorbance of FTRNPs solutions at 650 nm increased quite linearly with the H_2_O_2_ concentration in the range of 0 − 80 µM (Fig. 3e). After co-incubation with different concentrations of H_2_O_2_, the color evolution was recorded (Fig. 3f).

The significant absorbance of oxidized TMB in the NIR region made FTRNPs a good candidate for PAI. In vitro PA images of FTRNPs solution in “on” (after H_2_O_2_ treatment) or “off” (before H_2_O_2_ treatment) states were recorded from 690 to 990 nm (Fig. 3 g). Their PA spectra presented obvious differences that were basically consistent with the absorption spectra (Fig. 3 h). At the same time, PA images of FTRNPs solution after co-incubation with different concentrations of H_2_O_2_ indicated that the increase of PA amplitude was related to the H_2_O_2_ concentration (Fig. 3i, j). The aforementioned results demonstrated that Fe_3_O_4_ mediated Fenton reaction and TMB oxidation endowed FTRNPs with the capability of tumor-specific in situ PAI. The cascade “turn on” process by the TME held a promising potential for improving the specificity of tumor PAI.

Photothermal capability of FTRNPs was demonstrated in vitro. At different pH levels, FTRNPs treated with H_2_O_2_ were irradiated by a NIR laser (wavelength: 808 nm, intensity: 1 W/cm^2^, time: 10 min), using FTRNPs without H_2_O_2_-treatment as a control (Fig. S3). For H_2_O_2_-treated samples, the heating rate was obviously related to the pH level. The temperature of FTRNPs in mild acid conditions (pH = 6.0) gradually increased and achieved a maximum value at 72.6 °C, while the temperature of that in neutral conditions (pH = 7.4) was just slowly increased to 51.5 °C. In contrast, the temperature of FTRNPs in alkaline conditions (pH = 9.6) basically did not increase. For samples without H_2_O_2_-treatment, the temperature before and after laser irradiation had little change at all pH values (Fig. S4). Moreover, the FTRNPs after H_2_O_2_ treatment exhibited superior photothermal stability (Fig. S5) and high photothermal conversion efficiency (η = 50.9%). Such a H_2_O_2_ and acidity dual-responsive nanoplatform had great potential in further application for tumor-specific PAI-guided PTT.

### In vitro FTRNPs mediated cytotoxicity profiles

**Fig. 4:**
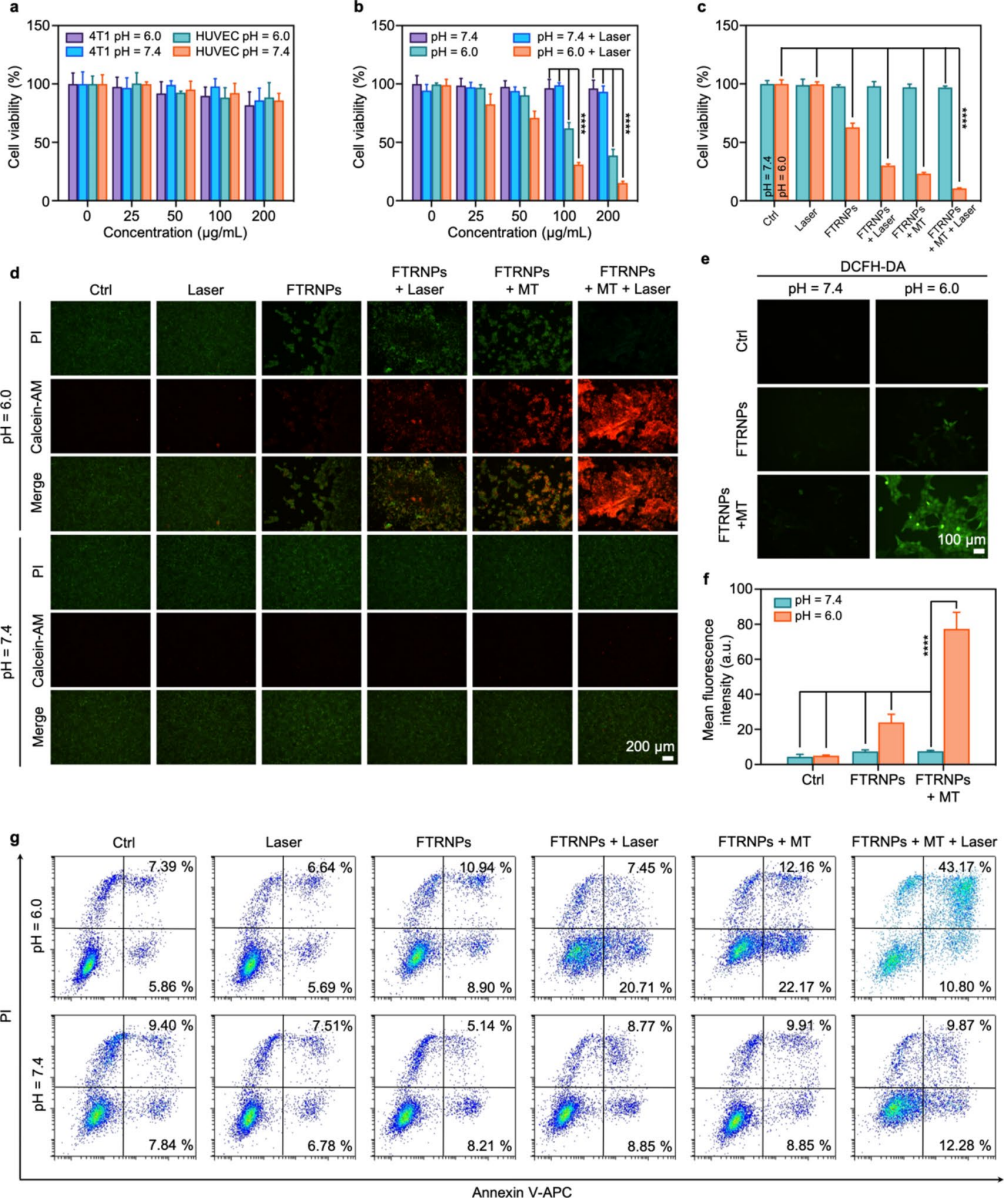
Synergistic CDT/PTT in vitro. (a) Biocompatibility of FTRNPs at mild acidic conditions (pH = 6.0) and neutral conditions (pH = 7.4) without H_2_O_2_. (b) Synergistic CDT/PTT cytotoxicity of FTRNPs at mild acidic conditions (pH = 6.0) and neutral conditions (pH = 7.4) with H_2_O_2_ (50 µM). Cell viability (c) and Calcein-AM/PI staining (d) of 4T1 cells with different treatments (PBS/FTRNPs (100 µg/mL, Fe_3_O_4_), mild acidic conditions/ neutral conditions, with/without irradiation (808 nm, 1 W/cm^2^, 5 min), with/without magnetic targeting) with H_2_O_2_ (50 µM). (e) FL images of 4T1 cells incubated with DCFH-DA probe under different conditions (PBS/FTRNPs (100 µg/mL, Fe_3_O_4_), mild acidic conditions/neutral conditions, with/without magnetic targeting). (f) Quantification of DCFH-DA probe after different treatments. (g) Flow cytometric apoptosis/necrosis analysis based on Annexin V-APC/PI staining assay of 4T1 cells with different treatments (PBS/FTRNPs (100 µg/mL, Fe_3_O_4_), mild acidic conditions/ neutral conditions, with/without irradiation (808 nm, 1 W/cm^2^, 5 min), with/without magnetic targeting).

Before studying the synergistic anticancer effect, the biocompatibility of FTRNPs was investigated in vitro. FTRNPs at different concentrations were co-incubated with human umbilical vein endothelial cells (HUVEC) or murine mammary carcinoma cells (4T1) without H_2_O_2_ treatment (n = 4). The cellular viabilities were detected *via* a thiazolyl blue tetrazolium bromide (MTT) assay after 24 h of co-incubation with FTRNPs. No obvious toxicity was observed from the result, indicating the ideal biocompatibility of FTRNPs (Fig. 4a). In addition, in order to simulate the physicochemical conditions of healthy tissues and tumorous tissues, neutral conditions (pH = 7.4) and mild acid conditions (pH = 6.0) were used in biocompatibility studies. The results showed that the effect of mild acidic conditions on cell viability of tumor cells was negligible.

Killing cancer cells with •OH generated by Fenton reaction of Fe_3_O_4_ NPs is an effective CDT strategy. At the same time, oxidized TMB is an efficacious PTT agent due to its excellent photothermal conversion properties. In this way, FTRNPs at the “on state” was well-suited for synergistic CDT/PTT. Synergistic CDT/PTT cytotoxicity was then investigated quantitatively by MTT assay (Fig. 4b). 4T1 cells with H_2_O_2_ (50 µM) were treated with FTRNPs in gradient concentrations in both neutral conditions (pH = 7.4) and mild acid conditions (pH = 6.0) for 8 h (n = 4). Then NIR laser (wavelength: 808 nm, intensity: 1.0 W/cm^2^, time: 5 min) treatment was used to realize PTT. Subsequently, the cellular viabilities of all groups were detected at 16 h after different treatments. Under neutral conditions (pH = 7.4), the cells maintained high cell viability whether laser irradiation was applied or not, indicating FTRNPs at “off state” had no obvious cytotoxicity due to the absence of Fe_3_O_4_ mediated Fenton reactions and TMB oxidation. In contrast, FTRNPs with H_2_O_2_ treatment exhibited cytotoxicity at mild acidic conditions (pH = 6.0). Furthermore, the oxidized TMB mediated PTT further reduced cell viability for the pH = 6.0 group. Therefore, synergistic CDT/PTT capabilities of FTRNPs can be specifically activated in TME.

In vitro anticancer ability of FTRNPs was evaluated by both MTT assay and living and dead cell co-staining (Fig. 4c, d). All groups (n = 4) were treated with H_2_O_2_ (50 µM). At the same time, considering the magnetic properties of FTRNPs, magnetic targeting was adopted to enhance the drug uptake ability through the magnetic promotion. The results revealed that both laser treatment and magnetic targeting could enhance the anticancer ability of FTRNPs under mild acid conditions (pH = 6.0), while neither laser treatment nor magnetic targeting could enhance the ability under neutral conditions (pH = 7.4). It was evident that the combination of laser treatment and magnetic targeting could achieve the maximum anticancer ability. Under mild acid conditions (pH = 6.0), the cell viability of the group treated with FTRNPs with laser treatment and magnetic targeting (10.59 ± 0.43%) was only 16.85% of that without laser treatment and magnetic targeting (62.85 ± 3.54%).

In order to study the intracellular mechanism of FTRNPs, 2’,7’-dichlorofuorescin diacetate (DCFH-DA) was applied to quantitatively determine the hydroxyl radical produced intracellularly via FTRNPs at the “on state” (Fig. 4e, f). Non-fluorescent DCFH-DA can be oxidized by •OH in cells to produce fluorescent 2’,7’- dichloro-fluorescein (DCF). All groups (n = 4) were treated with H_2_O_2_ (100 µM). There was almost no fluorescence in the group under neutral conditions (pH = 7.4), indicating that mild acidic environment was an important prerequisite for triggering the Fenton reaction of Fe_3_O_4_ NPs. For mild acid conditions (pH = 6.0) groups, the mean fluorescence intensity of the group with magnetic targeting (77.29 ± 9.55) was 3.21-fold higher than the group without magnetic targeting (24.05 ± 4.62) and 15.27-fold higher than the control group (5.03 ± 0.40). The results demonstrated that FTRNPs could generate •OH under mild acid conditions (pH = 6.0) in the presence of H_2_O_2_. Meanwhile, magnetic targeting could effectively enhance the drug uptake by tumor cells and increase the production of •OH, so as to significantly improve the effect of CDT.

Furthermore, the apoptosis characteristics of 4T1 cells after different treatments (PBS/FTRNPs (100 µg/mL, Fe_3_O_4_), mild acidic conditions/neutral conditions, with/without irradiation (808 nm, 1 W/cm^2^, 5 min), with/without magnetic targeting) were studied by flow cytometry (Fig. 4 g, Fig. S6). Cells of all groups were labeled by apoptosis kit (Annexin V-APC/PI) after different treatments. For the mild acid conditions (pH = 6.0) groups, the groups with laser treatment or magnetic targeting displayed much higher apoptosis, including early and late apoptosis, compared with the group only treated with FTRNPs. Meanwhile, the FTRNPs-treated groups with both laser treatment and magnetic targeting showed ~ 53.97% apoptosis. In contrast, all treated groups at the neutral conditions (pH = 7.4) showed negligible effect on the cell apoptosis. These results further identified the tumor-specific photo-chemodynamic synergy effect *via* dual-targeting nanozyme.

### In vivo biocompatibility of FTRNPs

Hematology analysis, blood biochemistry, and histological examination (H&E) were applied for evaluating in vivo biocompatibility of FTRNPs. After systemic administration of FTRNPs (10 mg/kg, Fe_3_O_4_, n = 3), the main parameters of blood biochemistry and hematology, including white blood cell counts (WBC), red blood cell counts (RBC), granulocyte percentage (Gran), hemoglobin (HGB), hematocrit (HCT), mean corpuscular hemoglobin concentration (MCHC), mean platelet volume (MPV), mean corpuscular volume (MCV), platelets (PLT), red blood cell distribution width (RDW), lymphocyte percentage (Lymph), monocyte percentage (Mon), showed no apparent abnormalities on days 1, 7 and 14, comparing with those of the control group (Fig. S7). Moreover, the H&E results of major organs (including heart, liver, spleen, lung, and kidney) of FTRNPs-treated mice (10 mg/kg, Fe_3_O_4_) showed no obvious histopathological abnormalities or lesions on days 1, 7, and 14 (Fig. S8). Thus, these results indicated that there were no FTRNPs-induced toxicities in vivo.

### In vivo dual-responsive tumor-specific imaging

The PAI capability of our intelligent nanoplatform was first validated by intratumoral injection experiments. FTNPs (25 µL, 1 mg/mL, Fe_3_O_4_) was injected into the 4T1 xenograft tumors (n = 3) implanted subcutaneously on the back of the mice (BALB/c). The PA images were recorded at 690 nm at 0 h and 4 h after FTNPs-treatment via a homemade PACT system (Fig. S9). The PA amplitude at the tumor sites at 4 h after FTNPs-treatment (24.85 ± 1.01) was 2.92-times higher than that before FTNPs-treatment (8.52 ± 0.90). To further prove the capability of our intelligent nanoplatform for tumor imaging in situ, Cy5.5-labeled-FTNPs (10 mg/kg, Fe_3_O_4_) was intravenously injected into tumor-bearing BALB/c mice (n = 3). Then, fluorescence (FL) images were recorded via in vivo imaging system (IVIS) at 0, 4, 8, 12, and 24 h post-injection (Fig. S10). Due to the passive accumulation of FTNPs in the tumor through the enhanced permeability and retention (EPR) effect, the fluorescence intensity at the tumor site reached the highest at about 4 h post-injection. The PA images of the tumor sites were also recorded after systemic administration of FTNPs (10 mg/kg, Fe_3_O_4_, n = 3). PA imaging achieved the unanimous results as FL imaging and reached the maximum PA amplitude at about 4 h post-injection (Fig. S11). At this time point, PA amplitude at the tumor sites (22.03 ± 2.35) was 3.54-fold higher than that at 0 h post-injection (6.22 ± 1.24). All these results indicating that our intelligent nanoplatform had ideal TME-response and PAI capability.

### In vivo dual-targeting tumor-specific imaging

**Fig. 5:**
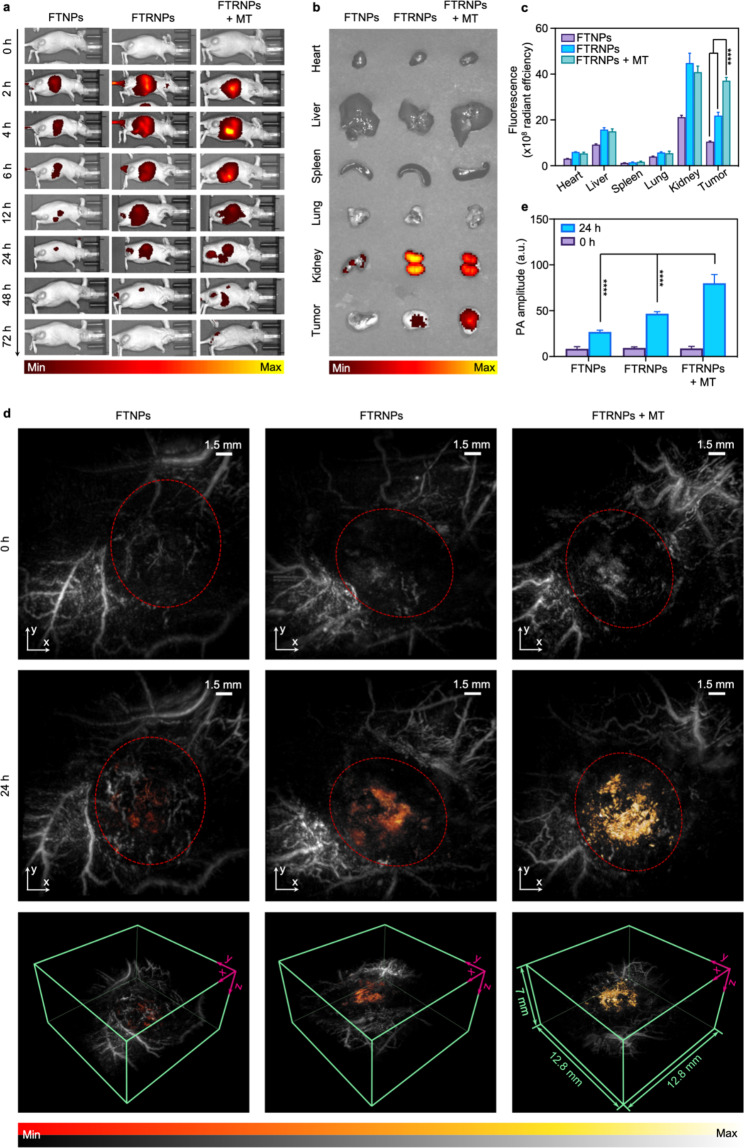
Multimodal imaging of FTRNPs in vivo. (a) In vivo FL imaging. FL images (b) of major organs and tumor of mice at 24 h post-injection of FTNPs/FTRNPs (10 mg/kg, Fe_3_O_4_) and the quantified results (c). (d) In vivo PAMe images (red dashed: tumor). (e) Quantification of the PA signals before and after systemic administration of FTNPs/FTRNPs (10 mg/kg, Fe_3_O_4_).

RGD peptides can specifically bind to the overexpressed αvβ3 integrin on tumor cell membrane. Therefore, the presence of the RGD peptide endowed FTRNPs the ability to actively target tumors. Besides, Fe_3_O_4_-based FTRNPs were suitable for magnetic targeting to enhance accumulation in tumors and magnetic promotion of drug uptake. To evaluate such in vivo dual-targeting ability of FTRNPs, FL imaging and PAI were conducted on 4T1 tumor-bearing mice (n = 3). For FL imaging, Cy5.5-labeled-FTRNPs (10 mg/kg, Fe_3_O_4_) was systemic administrated to demonstrate the active targeting (marked as FTRNPs). And another group Cy5.5-labeled-FTRNPs-treated mice (10 mg/kg, Fe_3_O_4_) were applied with a magnet placed at the tumor site for magnetic targeting to evaluate the dual-targeting ability (marked as FTRNPs + MT). Meanwhile, the group of Cy5.5-labeled-FTNPs-treated mice (10 mg/kg, Fe_3_O_4_) without RGD peptide was used to evaluate the EPR effect (marked as FTNPs). All groups were imaged at 0, 2, 4, 6, 12, 24, 48, and 72 h post-injection through IVIS (Fig. 5a). Both the FTRNPs group and the FTRNPs + MT group reached a peak at 24 h after systemic administration, while the FTNPs group showed relatively lower FL intensity at all detected time. Interestingly, compared with Fig. S10, the tumors of FTNPs group in Fig. 5a had almost no fluorescence signal, which might be caused by different color bars. This further indicated that the combined use of biological active targeting and physical active targeting substantially improved the accumulation and retention of FTRNPs at the tumor site. At the time point of 24 h, mice of all groups were killed and their tumors and major organs (heart, liver, spleen, lung, kidney) were separated and recorded by IVIS (Fig. 5b, c). For the FTRNPs + MT group, the ex vivo tumor FL intensity (37.17 ± 1.40) was 3.54-fold higher than the FTNPs group (10.51 ± 0.31) and 1.69-fold higher than the FTRNPs group (21.93 ± 1.41). Meanwhile, in the ex vivo main organs, the fluorescence signal was mainly concentrated in the kidney, indicating that FTRNPs were mainly eliminated through renal metabolism, which might be due to its small size. In addition, the wide range fluorescence signal in the abdomen within 12 h and the fluorescence signal in the isolated liver at 24 h indicated that the intestine and liver also participated in the metabolism of FTRNPs.

To further study the dual-responsive and dual-targeting ability of FTRNPs, three-dimensional in vivo PA images were acquired by PAMe. Same as FL imaging, mice treated with non-Cy5.5-labeled nanoplatforms (10 mg/kg, Fe_3_O_4_) were divided into FTNPs group, FTRNPs group, and FTRNPs + MT group (n = 3). PA images at the tumor site were imaged at 690 nm before and after systemic administration (24 h). The reconstructed tomograms were shown as maximum intensity projection images (Fig. 5d). Obvious PA signals from the intelligent nanoplatform appeared at the tumor sites. Moreover, both biologically active targeting of RGD and magnetic targeting could significantly enhance the PA signal at the tumor sites. More quantitatively, the average PA values at the tumor site of the FTRNPs + MT group (80.02 ± 9.50) and the FTRNPs group (46.70 ± 2.26) were 2.99 and 1.74 times that of the FTNPs group (26.80 ± 1.92), respectively (Fig. 5e).

In sum, all these results demonstrated that our intelligent nanoplatform FTRNPs can efficiently react with H_2_O_2_ in the mild acidic TME to produce detectable highly tumor-specific PA signal changes. Furthermore, both biologically and physically active targeting can evidently increase the accumulation of FTRNPs and promote the production of PAI/PTT agents (oxidized TMB), which might help to achieve highly specific and highly effective tumor theranostics.

### In vivo photo-chemodynamic therapy

**Fig. 6:**
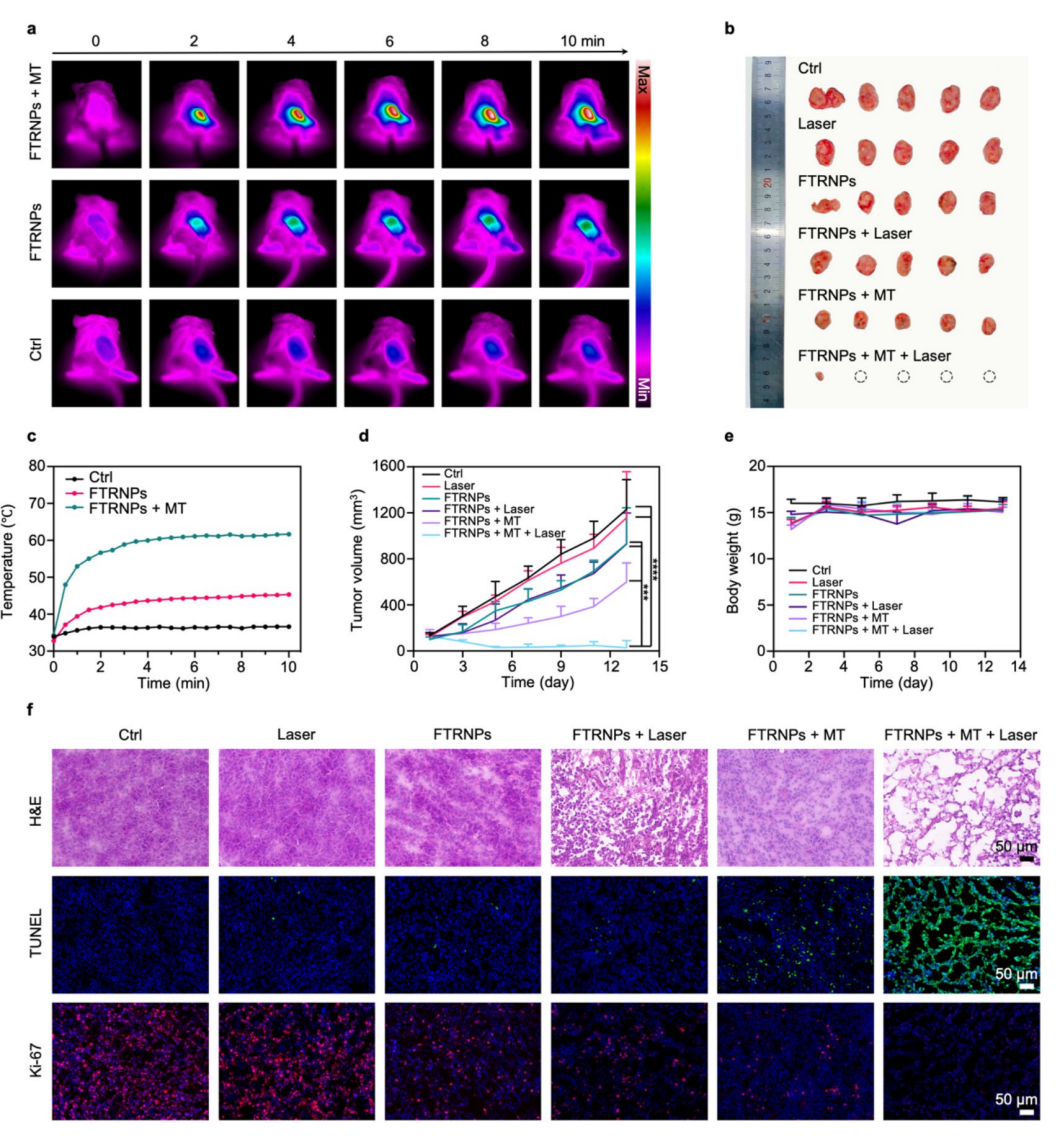
Synergistic CDT/PTT in vivo. (a) In vivo IR thermal images of tumor-bearing mice with different treatments (PBS/FTRNPs (100 µg/mL, Fe_3_O_4_), with/without magnetic targeting) under NIR laser irradiation. (b) Photograph of the tumors after different treatments (PBS/FTRNPs (100 µg/mL, Fe_3_O_4_), with/without irradiation (808 nm, 2 W/cm^2^, 10 min), with/without magnetic targeting). (c) Real-time temperature of tumor sites under NIR laser irradiation. Tumor growth curves (d) and body weights (e) of different groups after different treatments (PBS/FTRNPs (100 µg/mL, Fe_3_O_4_), with/without irradiation (808 nm, 2 W/cm^2^, 10 min). (f) Histopathological analysis of the excised tumor slices after different treatments (PBS/FTRNPs (100 µg/mL, Fe_3_O_4_), with/without irradiation (808 nm, 2 W/cm^2^, 10 min), including H&E staining, TUNEL assay and Ki-67 staining.

Before evaluating the antitumor effect in vivo, photothermal capability of FTRNPs was demonstrated in vivo. Mice were divided into three groups (n = 5): the FTRNPs group (10 mg/kg, Fe_3_O_4_), the FTRNPs + MT group (10 mg/kg, Fe_3_O_4_, with magnetic targeting), and the control group (phosphate-buffered saline (PBS) treatment). At 24 h post-injection, all groups were treated by a NIR laser (wavelength: 808 nm, intensity: 2 W/cm^2^, time: 10 min). Meanwhile, an infrared radiation thermal camera was applied to record real-time temperatures at 30-second intervals (Fig. 6a, c). The tumor temperature of the FTRNPs + MT group gradually increased and achieved the maximum at 61.7 °C, while the tumor temperature of the FTRNPs group was just slowly increased from body temperature to the maximum at 45.3 °C. In contrast, the tumor temperature of the control group basically did not increase. Meanwhile, benefited from the *in-situ* generation of photothermal agent and the accurate laser irradiation, there were no obvious damage on normal tissues around lesions during PTT, which was indicated by comparing the temperature of the tumor site with the temperature around the normal tissue (Fig. S12). These results demonstrated that FTRNPs can be used for specific PTT, and magnetic targeting can promote the efficient photothermal destruction of cancer cells.

The in vivo photo-chemodynamic therapy performance of FTRNPs was studied using 4T1 tumor-bearing mice. Six groups (n = 5) were set up in the experiment, including control, laser, FTRNPs, FTRNPs + laser, FTRNPs + MT, FTRNPs + MT + laser. Each FTRNPs treatment group received repeated systemic administrations on days 0, 3, 6, and 9 (10 mg/kg, Fe_3_O_4_). For those groups treated with magnetic targeting, an external magnetic field was applied at their tumor sites after every systemic administration for 24 h. For those “laser” groups, the tumor sites were treated with an irradiation (wavelength: 808 nm, intensity: 2 W/cm^2^, time: 10 min) at 24 h after the first systemic administration. Moreover, the tumor volumes were monitored every two days to evaluate the effect of the synergistic therapeutic effects (Fig. 6d). Compared with the control group and all laser-treated group, the tumor growth of those FTRNPs treated groups was inhibited to varying degrees, indicating that FTRNPs had a specific CDT effect. Quantitatively, on day 13 after treatment, the tumor volumes of “FTRNPs” group (930.57 ± 313.71), “FTRNPs + laser” group (928.64 ± 269.69), “FTRNPs + MT” group (599.12 ± 165.62) and “FTRNPs + MT + laser” (27.61 ± 61.74) group were 75.66%, 75.48%, 48.69%, and 2.24% of that of the control group (1230.38 ± 258.81), respectively. The tumor inhibition rate of photo-chemodynamic therapy was ~ 97.76%, which was ~ 4-fold higher than that of PTT or CDT only. Meanwhile, by comparing the FTRNPs + MT group and the FTRNPs + MT + laser group with other groups, it was proved that magnetic targeting can effectively improve the therapeutic effects of both CDT and PTT. On day 13 after treatment, the tumor volume of “FTRNPs + MT + laser” (27.61 ± 61.74) group were 2.97% of that of “FTRNPs + laser” group (928.64 ± 269.69). As expected, the mice treated with FTRNPs plus magnetic targeting and laser exhibited the strongest tumor inhibition, indicating the substantial antitumor therapeutic effect of synergistic CDT/PTT. In addition, the body weights (Fig. 6e) of the mice were recorded with no significant changes, indicating no obvious biotoxicity during the synergistic CDT/PTT process. Additionally, the morphology and weight of the resected tumors confirmed the above conclusions (Fig. 6b, Fig. S13).

To comprehensively understand the pathological variation and mechanism of synergistic CDT/PTT, pathological analysis, including H&E, Ki-67 staining, and terminal deoxynucleotidyl transferase-mediated deoxyuridine triphosphate nick end labeling (TUNEL) were carried out (Fig. 6f). For the three laser-treated groups, tumors were isolated at ~ 5 h after laser irradiation, while tumors were isolated at 24 h after the last systemic administration for other groups. The decrease of the number of tumor nuclei was consistent with the effect of tumor treatment, according to the results of H&E staining. Moreover, the tumor tissue of the FTRNPs + MT + laser group showed obvious shrinkage and injury, while the FTRNPs + laser group showed partial damage. For the TUNEL staining, apparent tumor apoptotic cells were observed in the groups treated with FTRNPs. Notably, the FTRNPs + MT + laser group showed the strongest green fluorescence, indicating the enhanced antitumor effect attributed by synergistic CDT/PTT. On the contrary, the FTRNPs + MT + laser group exhibited the weakest fluorescence in the Ki-67 staining results, indicating that the proliferation of tumor cells was seriously impaired after synergistic CDT/PTT. In addition, as shown in the H&E results, the FTRNPs + laser group and the FTRNPs + MT + laser group showed different degrees of damage, which might be induced by cell necrosis. Meanwhile, as shown in the TUNEL results, the FTRNPs + MT group and the FTRNPs + MT + laser group showed different degrees of cell apoptosis. The results showed that PTT mainly caused tumor cell necrosis, while CDT caused tumor cell apoptosis. Therefore, the combination of CDT/PTT could effectively improve the therapy efficiency.

## Conclusion

In conclusion, we have successfully developed a H_2_O_2_/acidity cascade-dual-responsive and RGD/magnetic dual-targeting nanoplatform (FTRNPs) for cancer treatment. The FTRNPs after H_2_O_2_ treatment exhibited superior photothermal stability and high photothermal conversion efficiency (η = 50.9%). The as-prepared FTRNPs exhibited high tumor accumulation in vivo, attributed to the synergistic effect of RGD and magnetic-active targeting, which was 3.54-fold higher than that without active targeting. High imaging and therapeutic specificities were achieved based on Fenton reactions and TMB oxidation, which were activated by elevated H_2_O_2_ and acid levels in tumors. The newly developed FTRNPs can significantly reduce the nonspecific signals and treatment side effects routinely encountered by the “always on, single-activated” theranostic agents. More importantly, the photo-chemodynamic therapy achieved noteworthy anticancer effects both in vitro and in vivo, exhibiting great potential in future clinical applications. The therapy efficiency of photo-chemodynamic therapy was ~ 97.76%, which was ~ 4-fold higher than that PTT or CDT only. Meanwhile, the FTRNPs had shown high biocompatibility. Given the “all-in-one” advantages of this nanoplatform, the PAI-guided synergistic CDT/PTT was expected to pave the way for future tumor diagnosis and treatment. Considering the penetration depth limitation of NIR-I and the limited H_2_O_2_ concentration in TME, NIR-II PTT and H_2_O_2_ enriched vector should be developed in the future.

## Experimental section

### Materials and reagents

Iron tri(acetylacetonate), oleic acid, and benzyl ether were purchased from Sigma-Aldrich. DMSO and TMB was purchased from Aladdin. DSPE-PEG2000 was purchased from Avanti-Polar. DSPE-PEG2000-cRGD was purchased from Xi’an RuiXi. DSPE-PEG2000-Cy5.5 was purchased from Xi’an QiYue. MTT was purchased from Beijing Soledad. DCFH-DA was purchased from Meilunbio. DMEM, fetal bovine serum (FBS), PBS and Roswell Park Memorial Institute 1640 medium (RPMI 1640) were brought from Gibico. De-ionized water was obtained by Milli-Q Gradient.

### Synthesis of FTRNPs/FTNPs

FTRNPs and FTNPs were prepared using a classical thin-film rehydration method. For FTRNPs, oleic acid modified Fe_3_O_4_ NPs (1 mg), TMB (0.1 mg), DSPE-PEG2000 (1.6 mg), and DSPE-PEG2000-cRGD (0.2 mg) were ultrasonically dispersed into 1mL of chloroform, followed by water bath ultrasound sonication for half hour. Subsequently, the mixture was evaporated to form a lipid film at 45 °C. Subsequently, the film was hydrated with PBS (1mL, mild acidic conditions or neutral conditions) and was treated by water bath ultrasound sonication for 20 min. The FTRNPs was washed by centrifugation for removing excess lipids. FTNPs were also prepared by the same method, using DSPE-PEG2000 of the same amount instead of DSPE-PEG2000-cRGD. The prepared FTRNPs and FTNPs were stored at 4 °C.

### Characterization of FTRNPs

Structures were observed via TEM (JEM-1200 EX, 120 kV). High-resolution TEM and element mapping were characterized by TEM (Tecnai, 300 kV). The hydrodynamic size and zeta-potential were recorded using a zetasizer (Nano ZS90, Malvern). XRD was recorded by an X-ray diffractometer (D-eight, ADVANCE). XPS spectra were measured using an X-ray photoelectron spectroscopy (250Xi, Thermo-Fisher). And the hysteresis curve was obtained via SQUID-VSM (MPMS-3, Quantum Design).

### H_2_O_2_-responsiveness of FTRNPs

FTRNPs (0.5 mg/mL, Fe_3_O_4_) under mild acidic conditions (pH = 6.0) was treated with different concentrations of H_2_O_2_ for 1 h. Then, the absorbances were measured by a UV − vis spectrophotometer (UV2300, Techcomp).

### pH-Responsiveness of FTRNPs

FTRNPs (0.5 mg/mL, Fe_3_O_4_) was treated with H_2_O_2_ (80 µM) at different pH levels for 60 min. Then, the absorbances were measured by a UV − vis spectrophotometer (UV2300, Techcomp).

### Photothermal conversion efficiency measurements

To evaluate the photothermal conversion efficiency, a FTRNPs solution (1 mg/mL, 3 mL) was heated to a steady temperature with an 808 nm laser at a power density of 1 W/cm^2^ and then naturally cooled. The photothermal conversion efficiency was calculated according to the previous literature [46].

### PA spectroscopic measurements

The PA spectra was measured by a home-made PACT system. A 532 nm laser was applied to pump and produce a laser pulse from 680 to 1064 nm. The ultrasound detector array (Imasonic Inc.) was used to detect the PA signals. FTRNPs (0.5 mg/mL, Fe_3_O_4_) after treated with H_2_O_2_ (different concentrations) were placed inside a polytetrafluoroethylene (PTFE) tube. The PTFE tubes were irradiated with laser (wavelength: 680–990 nm, increment: 10 nm). Then, the PA spectra was recorded by from 680 to 990 nm by normalized the peak-to-peak voltage of the PA signal.

### Photothermal properties

FTRNPs (0.5 mg/mL, Fe_3_O_4_) at different pH levels (pH = 6.0, 7.4, 9.6) were divided into two groups. The first group was incubated with H_2_O_2_ (80 µM) for 60 min, while the second group was incubated with PBS. All samples were irradiated by a laser (wavelength: 808 nm, intensity: 1 W/cm^2^) for 10 min.

### In vitro biocompatibility of FTRNPs

Both 4T1 cells and HUVECs were inoculated in 96-well plates and divided into two groups. One group was incubated under mild acidic conditions (pH = 6.0), while the other group was under neutral conditions (pH = 7.4). Cells were treated with FTRNPs (0-200 µg/mL, Fe_3_O_4_) for 1 day. Subsequently, the biocompatibilities were evaluated by MTT assay.

### *In vitro* cytotoxicity assay of FTRNPs

To test the CDT induced cytotoxicity from Fe_3_O_4_ Fenton reaction, 4T1 cells in 96 well plates were divided into two groups. One group was incubated under mild acidic conditions (pH = 6.0), while the other was under neutral conditions (pH = 7.4). Cells were treated with FTRNPs (0-200 µg/mL, Fe_3_O_4_) and H_2_O_2_ (50 µM) for 1 day, while the control group was treated with PBS. Then, the cytotoxicity was evaluated by MTT assay. To measure the PTT-induced cytotoxicity, two groups of 4T1 cells were applied. One group was incubated under mild acidic conditions (pH = 6.0), while the other was under neutral conditions (pH = 7.4). Cells were co-incubated with FTRNPs (H_2_O_2_ preincubated, 0-200 µg/mL, Fe_3_O_4_) and H_2_O_2_ (50 µM) for 8 h. Subsequently, cells were treated with laser irradiation (wavelength: 808 nm, intensity: 1 W/cm^2^, time: 5 min). Then, the cells were further cultured for 16 h after washing by PBS. Subsequently, the cytotoxicity was evaluated by MTT assay. The non-irradiated PBS-treated cells were used as a control. To test the synergistic CDT/PTT induced cytotoxicity, both MTT and Calcein-AM/PI assays were carried out. 4T1 cells were divided into 12 groups: (1) pH = 6.0 + H_2_O_2_; (2) pH = 6.0 + H_2_O_2_ + laser; (3) pH = 6.0 + H_2_O_2_ + FTRNPs; (4) pH = 6.0 + H_2_O_2_ + FTRNPs + laser; (5) pH = 6.0 + H_2_O_2_ + FTRNPs + magnetic targeting; (6) pH = 6.0 + H_2_O_2_ + FTRNPs + magnetic targeting + laser; (7) pH = 7.4 + H_2_O_2_; (8) pH = 7.4 + H_2_O_2_ + laser; (9) pH = 7.4 + H_2_O_2_ + FTRNPs; (10) pH = 7.4 + H_2_O_2_ + FTRNPs + laser ; (11) pH = 7.4 + H_2_O_2_ + FTRNPs + magnetic targeting; (12) pH = 7.4 + H_2_O_2_ + FTRNPs + magnetic targeting + laser, while cells only with PBS-treated were used as a control. For “laser”, parameters (wavelength: 808 nm, intensity: 1 W/cm^2^, time: 5 min) were used. For “FTRNPs”, concentration (100 µg/mL, Fe_3_O_4_) was used. For “H_2_O_2_”, concentration (50 µM) was used. After treatment, the cytotoxicity was recorded by MTT assay. For “magnetic targeting”, circular permanent magnets were placed below 96 well plates for 8 h. For living and dead cell staining assay, 4T1 cells in 12 well plates were divided into 12 groups as the MTT assay. The treated cells were stained using Calcein-AM and PI for half hour before FL imaging. Then, an inverted FL microscope (Leica DMi8) was used to observe the stained cells.

### *In vitro* •OH generation of FTRNPs

4T1 cells were seeded in 12 well plates and divided into 6 groups: (1) pH = 6.0 + H_2_O_2_; (2) pH = 6.0 + H_2_O_2_ + FTRNPs; (3) pH = 6.0 + H_2_O_2_ + FTRNPs + magnetic targeting; (4) pH = 7.4 + H_2_O_2_; (5) pH = 7.4 + H_2_O_2_ + FTRNPs; (6) pH = 7.4 + H_2_O_2_ + FTRNPs + magnetic targeting. For “FTRNPs”, concentration (100 µg/mL, Fe_3_O_4_) was used. For “H_2_O_2_”, concentration (50 µM) was used. For “magnetic targeting”, circular permanent magnets were placed below 12 well plates for 8 h. After 8 h, the 4T1 cells were treated with laser irradiation (wavelength: 808 nm, intensity: 1 W/cm^2^, time: 5 min). Then, the cells were washed with PBS and further cultured for 16 h. DCFH-DA (10 µM) was applied for staining the treated cells for half hour before FL imaging. Then, an inverted FL microscope (Leica DMi8) was used to record the stained cells.

### *In vitro* apoptosis evaluation

Cells (4T1) in 12 well plates were divided into 12 groups: (1) pH = 6.0 + H_2_O_2_; (2) pH = 6.0 + H_2_O_2_ + laser; (3) pH = 6.0 + H_2_O_2_ + FTRNPs; (4) pH = 6.0 + H_2_O_2_ + FTRNPs + laser; (5) pH = 6.0 + H_2_O_2_ + FTRNPs + magnetic targeting; (6) pH = 6.0 + H_2_O_2_ + FTRNPs + magnetic targeting + laser; (7) pH = 7.4 + H_2_O_2_; (8) pH = 7.4 + H_2_O_2_ + laser; (9) pH = 7.4 + H_2_O_2_ + FTRNPs; (10) pH = 7.4 + H_2_O_2_ + FTRNPs + laser ; (11) pH = 7.4 + H_2_O_2_ + FTRNPs + magnetic targeting; (12) pH = 7.4 + H_2_O_2_ + FTRNPs + magnetic targeting + laser, while cells only treated with PBS were used as a control. For “laser”, parameters (wavelength: 808 nm, intensity: 1 W/cm^2^, time: 5 min) were used. For “FTRNPs”, concentration (100 µg/mL, Fe_3_O_4_) was used. For “H_2_O_2_”, concentration (50 µM) was used. For “magnetic targeting”, circular permanent magnets were placed below 12 well plates for 8 h. After different treatments, the cytotoxicity was recorded by MTT assay. Apoptosis Kit with Annexin V-APC and PI was used to assess the apoptosis by flow cytometer (FAC SAria II) at 12 h after the treatments.

### Animals and tumor models

BALB/C mice and BALB/C nude mice (female, 6-week-old) were provided by Beijing Vital River Laboratory Animal Technology Co., Ltd. All animal experiments were conducted in accordance with the regulations of the LARC of Tsinghua University in Beijing, China. To establish subcutaneous tumors, 4T1 cells (5 × 10^7^) were hypodermic injected in the backside of the mice.

### In vivo biocompatibility of FTRNPs

To evaluate in vivo biocompatibility, systemic administration of FTRNPs (10 mg/kg, Fe_3_O_4_) was applied on BALB/C nude mice (n = 3). At 0, 1, 7, and 14 days after systemic administration, a blood routine examination was performed. Meanwhile, the in vivo biocompatibility of FTRNPs was further assessed by a histological examination. Systemic administration of FTRNPs (10 mg/kg, Fe_3_O_4_) was applied on BALB/C nude mice (n = 3). At 0, 1, 7, and 14 days after systemic administration, major organs of the mice were removed and stained with H&E.

### *In vivo* FL imaging

For labeling Cy5.5, a small amount of DSPE-PEG-Cy5.5 (5% of phospholipid) was added during samples fabrication. For in vivo dual-responsive measurement, 6-week-old female 4T1-tumor-bearing nude mice (n = 3) were treated with Cy5.5-labeled-FTNPs (10 mg/kg, Fe_3_O_4_). At 0, 4, 8, 12, and 24 h after systemic administration, the FL images were recorded by IVIS. For in vivo dual-targeting measurement, 6-week-old female nude mice (n = 3) were treated with Cy5.5-labeled-FTRNPs (10 mg/kg, Fe_3_O_4_) and divided into 2 groups: (1) FTRNPs; (2) FTRNPs + magnetic targeting, while mice treated with Cy5.5-labeled-FTNPs (10 mg/kg) were used as a control. For magnetic targeting, circular permanent magnets were fixed to the surface of the tumors. At 0, 2, 4, 6, 12, 24, 48, and 72 h after systemic administration, the FL images were recorded by IVIS. Moreover, the major organs and tumors were collected and imaged at 24 h after systemic administration.

### In vivo PACT imaging

In vivo PA images were recorded by a homemade PACT system. A 532 nm laser, an optical parametric oscillator, and a full-ring ultrasound detector array were employed for PACT imaging. For intratumoral injection experiments, 6-week-old female nude mice (n = 3) were anesthetized using 2% isoflurane in oxygen. Tumors were injected with FTNPs (10 mg/kg, Fe_3_O_4_). For the tail vein injection experiment, female 4T1 tumor-bearing mice (n = 3) were anesthetized using 2% isoflurane in oxygen, and were imaged at 0, 4, 8, 12, and 24 h after systemic administration.

### *In vivo* PAMe imaging

In vivo PA images were recorded by a homemade PAMe system. A high-precision F-P interference sensor was employed for PAMe imaging. Images reconstruction was subsequently performed in three-dimensional. 4T1 tumor-bearing mice (n = 3) were treated with FTRNPs (10 mg/kg, Fe_3_O_4_) and divided into 2 groups: (1) FTRNPs; (2) FTRNPs + magnetic targeting, while mice treated with FTNPs (10 mg/kg, Fe_3_O_4_) were used as a control. For magnetic targeting, circular permanent magnets were fixed to the surface of the tumors. At 0 and 24 h after systemic administration, the PAMe images were taken at 690 nm.

### *In vivo* thermal imaging

Mice were treated with FTRNPs (10 mg/kg, Fe_3_O_4_) and divided into 2 groups (n = 3): (1) FTRNPs; (2) FTRNPs + magnetic targeting, while mice treated with PBS were used as a control. For magnetic targeting, circular permanent magnets were fixed to the surface of the tumors. All mice were treated by laser irradiation (wavelength: 808 nm, intensity: 2 W/cm^2^, time: 10 min) at 24 h after systemic administration. The thermal images and the temperature were recorded.

### *In vivo* synergistic CDT/PTT

Tumor-bearing mice were divided into 6 groups (n = 5) when the tumor volume was ~ 100 mm^3^: (1) PBS (as control); (2) PBS + laser; (3) FTRNPs; (4) FTRNPs + laser; (5) FTRNPs + magnetic targeting; (6) FTRNPs + magnetic targeting + laser. For “laser”, parameters (wavelength: 808 nm, intensity: 2 W/cm^2^, time: 10 min) were used. For “FTRNPs”, concentration (10 mg/kg, Fe_3_O_4_) was used. For magnetic targeting, circular permanent magnets were fixed to the surface of the tumors. Then, the mice began to receive different treatments on days 0, 3, 6 and 9. The tumor volumes of different groups were recorded and calculated every 2 days (V = L*W^2^/2). At the end of the therapeutic period, all tumors were removed, weighed and photographed.

### Pathological investigation

After in vivo treatment, tumors of all groups were collected and frozen with liquid nitrogen for histopathological analysis (H&E, TUNEL, Ki-67). For the laser-treated groups, tumors were removed at ~ 5 h after laser irradiation. For other groups, tumors were removed at 24 h after the last systemic administration.

### Image reconstruction

For PACT imaging, respiratory gating was used to average 131 frames of data. PACT images were reconstructed by adopting a delay-and-sum (DAS) method. For PAMe imaging, a three-dimensional DAS method was used to reconstruct the three-dimensional PAMe images. Hilbert transform and dual-speed-of-sound were employed during image reconstruction. All PACT and PAMe reconstructed images were rendered and displayed using MATLAB R2020b. Further image processing, including fluence compensation, image segmentation, and agent recognition, was performed with MATLAB.

### Statistical analysis

MATLAB R2020b was used to analysis region of interest (ROI) for measuring the PA signals. All data were presented as mean ± standard deviation (SD). GraphPad Prism 9 was used for statistical calculations. **p < 0.01, ***p < 0.001 and ****p < 0.001 were considered to be remarkable significant.

## Associated content

### Supporting information

Additional TEM Fig. for further characterization of Fe_3_O_4_ NPs. (Fig. S1); Photographs depict FTRNPs before and after magnetic field attraction. (Fig. S2); In vitro IR thermal images of FTRNPs. (Fig. S3); Real-time temperature of FTRNPs solution under NIR laser irradiation. (Fig. S4); Photostability study of FTRNPs (Fig. S4); Quantitative analysis of flow cytometric apoptosis/necrosis analysis. (Fig. S6); Complete blood panel and blood biochemistry analysis. (Fig. S7); H&E-stained tissue sections of major organs of mice receiving intravenous injection of FTRNPs. (Fig. S8); Additional PA images for further study the responsiveness of FTNPs. (Fig. S9, S11); Additional FL images for study the tumor accumulation of FTNPs. (Fig. S10); The temperature of the tumor site and the temperature around the normal tissue after irradiation (Fig. S12); Tumor weights of mice after different treatments. (Fig. S13).

## Electronic supplementary material

Below is the link to the electronic supplementary material.

Supplementary Material 1

## Data Availability

All data generated or analyzed during this study are included in this published article and its Additional files.
