# Supplementary material for: Dual-targeting nanozyme for tumor activatable photo-chemodynamic theranostics
Source: J Nanobiotechnology. 2022 Nov 3;20:466. doi: 10.1186/s12951-022-01662-9 (PMC9632160; doi:10.1186/s12951-022-01662-9)
Supplement: Supplementary file 1 — Supplementary Material 1 [file 12951_2022_1662_MOESM1_ESM.docx]

**Supporting Information**

**Dual-targeting nanozyme for tumor activatable photo-chemodynamic theranostics**

Chaoyi Chen^1, 2^, Yuwen Chen^2^, Lulu Zhang^1^, Xuanhao Wang^2^, Qingshuang Tang^1^, Yan Luo^2^, Yuan Wang^1^, Cheng Ma^2,3^* and Xiaolong Liang^1^*

^1^ Department of Ultrasound, Peking University Third Hospital, Beijing 100191, China

Add: 49 North Garden Rd., Haidian District Beijing, P.R. China

^2^ Department of Electronic Engineering, Beijing National Research Center for Information Science and Technology, Tsinghua University, Beijing 100084, China

^3^ Institute for Precision Healthcare, Tsinghua University, Beijing 100084, China

***Corresponding authors**

Xiaolong Liang ([xiaolong_liang@bjmu.edu.cn](mailto:xiaolong_liang@bjmu.edu.cn))

Cheng Ma (cheng_ma@tsinghua.edu.cn)


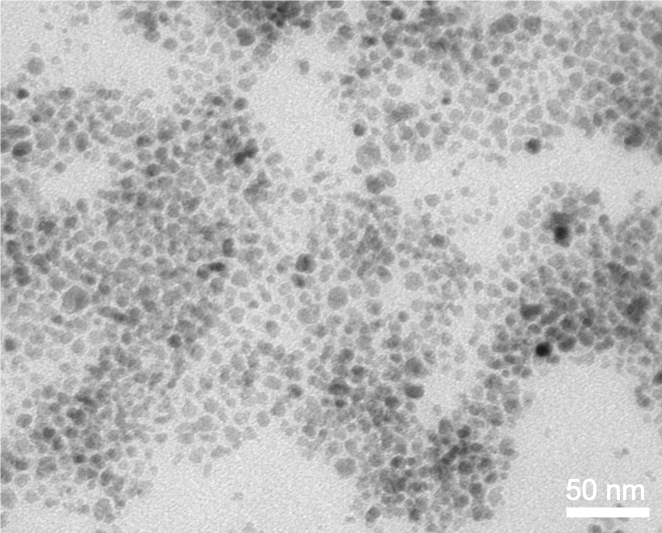


**Fig. S1** TEM image of Fe_3_O_4_ NPs.


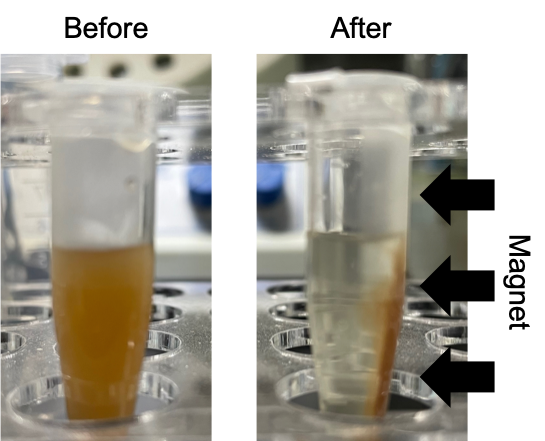


**Fig. S2** Photographs depict FTRNPs before and after magnetic field attraction.


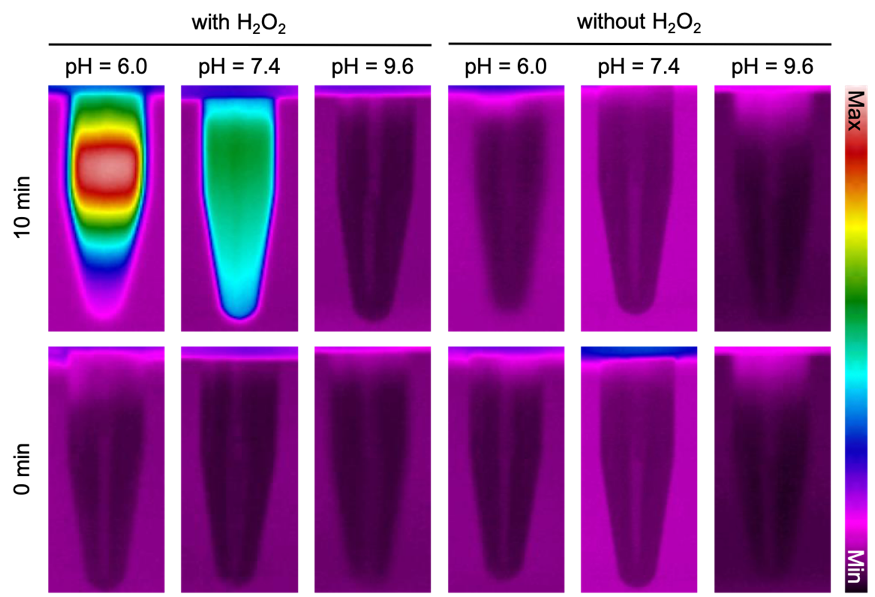


**Fig. S3** In vitro IR thermal images of FTRNPs (0.5 mg/mL, Fe_3_O_4_) with different treatment (H_2_O_2_, 80 μM, 60 min, pH 6.0-9.6) under NIR laser irradiation (808 nm, 1 W/cm^2^, 10 min).


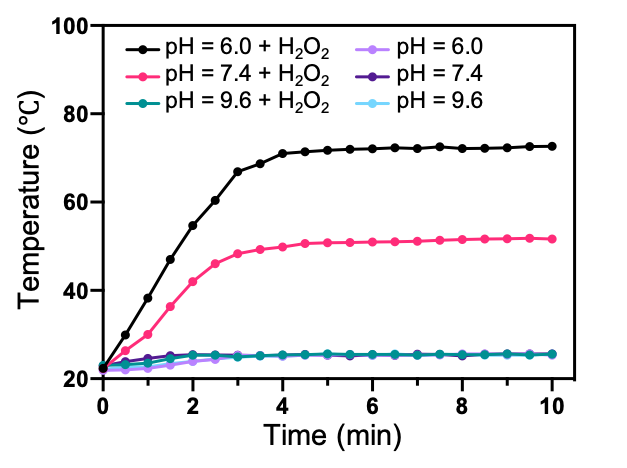


**Fig. S4** Real-time temperature monitoring of FTRNPs solution (0.5 mg/mL, Fe_3_O_4_) with different treatments (H_2_O_2_, 80 μM, 60 min, pH 6.0-9.6) under NIR laser irradiation (808 nm, 1 W/cm^2^).


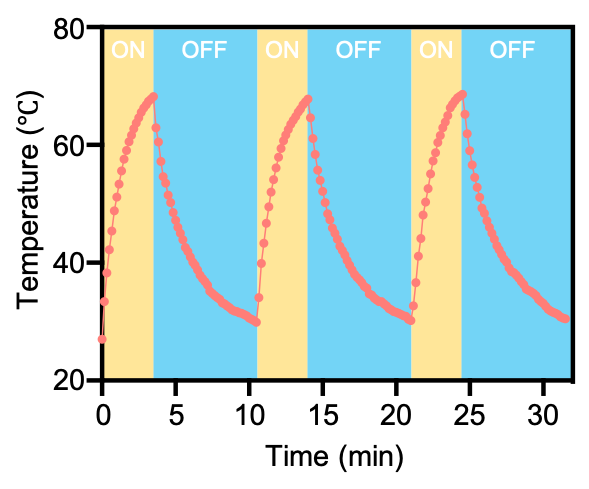


**Fig. S5** Photostability study of FTRNPs after H_2_O_2_ (80 μM) response in pH 6.0 PBS buffer by reversible photothermal heating (808 nm, 1 W/cm^2^) and natural cooling cycles.


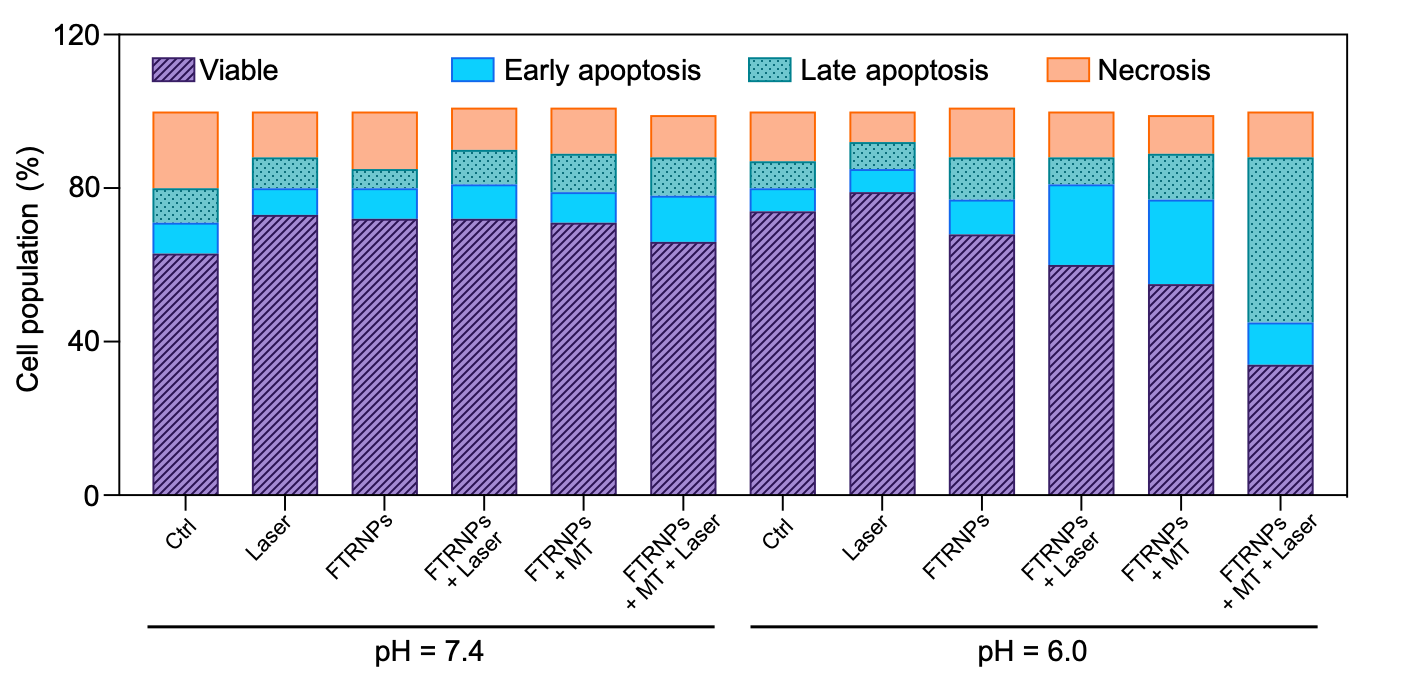


**Fig. S6** Quantitative analysis of flow cytometric apoptosis/necrosis analysis based on Annexin V-APC/PI staining assay of 4T1 cells with different treatments (PBS/FTRNPs (100 μg/mL, Fe_3_O_4_), mild acidic conditions/neutral conditions, with/without irradiation (808 nm, 1 W/cm^2^, 5 min), with/without magnetic targeting).


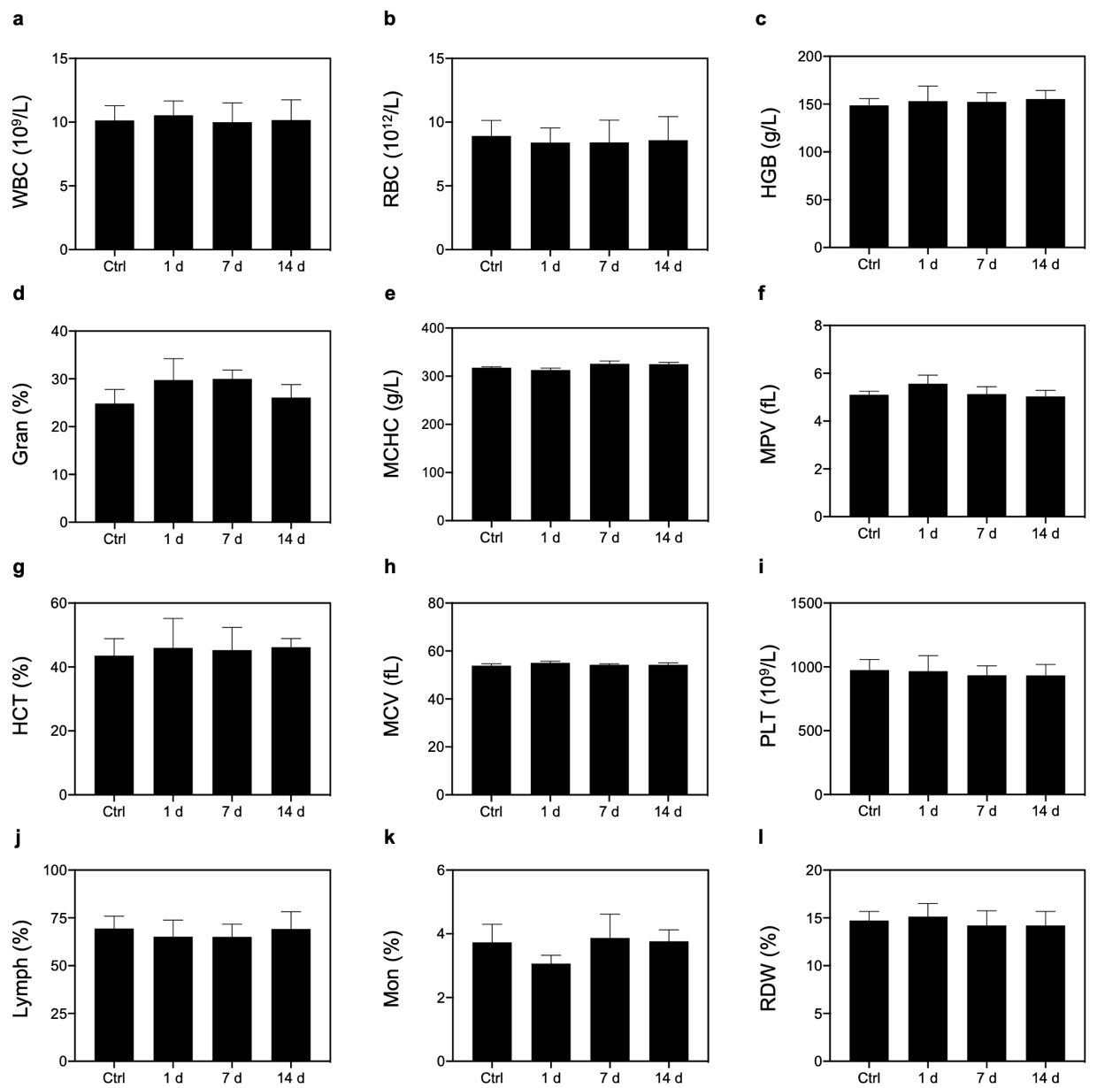


**Fig. S7** Complete blood panel and blood biochemistry analysis of mice *i. v.* injected with FTRNPs (10 mg/kg, Fe_3_O_4_). (a) White blood cell (WBC) counts. (b) Red blood cell (RBC) counts. (c) Hemoglobin (HGB). (d) Granulocyte percentage (Gran). (e) Mean corpuscular hemoglobin concentration (MCHC). (f) Mean platelet volume (MPV). (g) Hematocrit (HCT). (h) Mean corpuscular volume (MCV). (i) Platelets (PLT). (j) Lymphocyte percentage (Lymph). (k) Monocyte percentage (Mon). (l) Red blood cell distribution width (RDW).


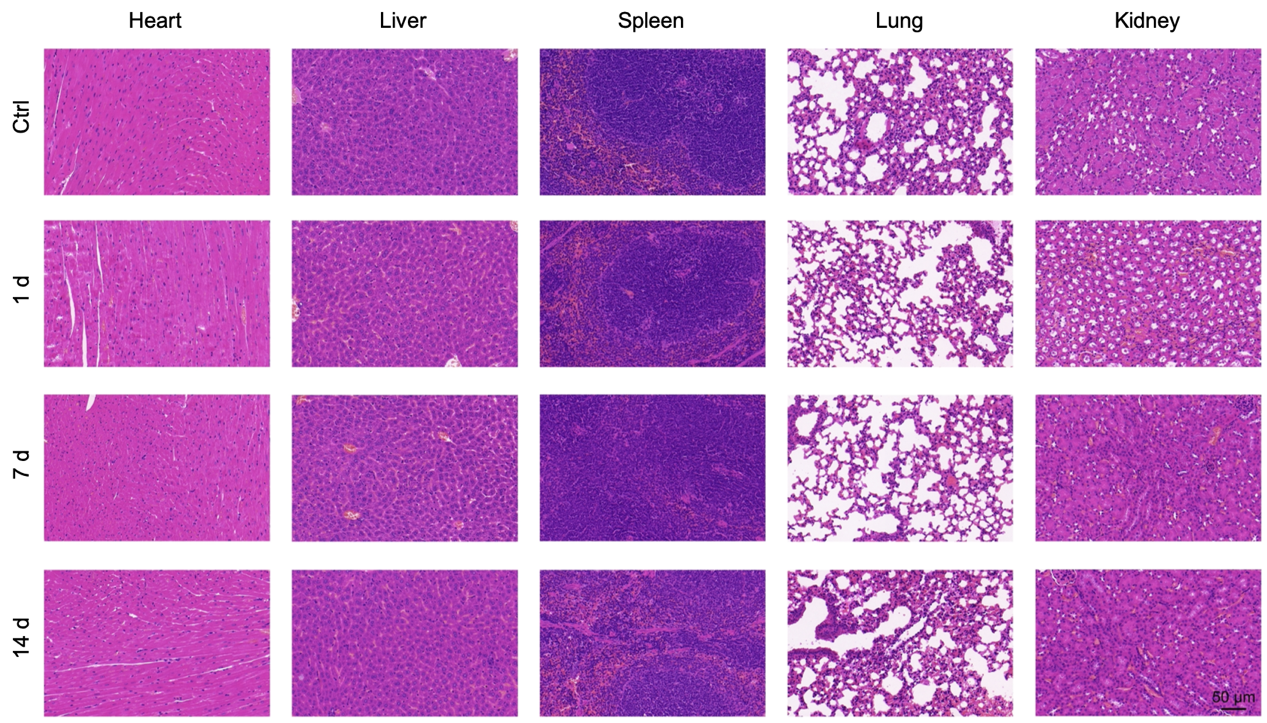


**Fig. S8** H&E-stained tissue sections of major organs including heart, liver, spleen, lung and kidney of mice receiving intravenous injection of FTRNPs (10 mg/kg, Fe_3_O_4_).


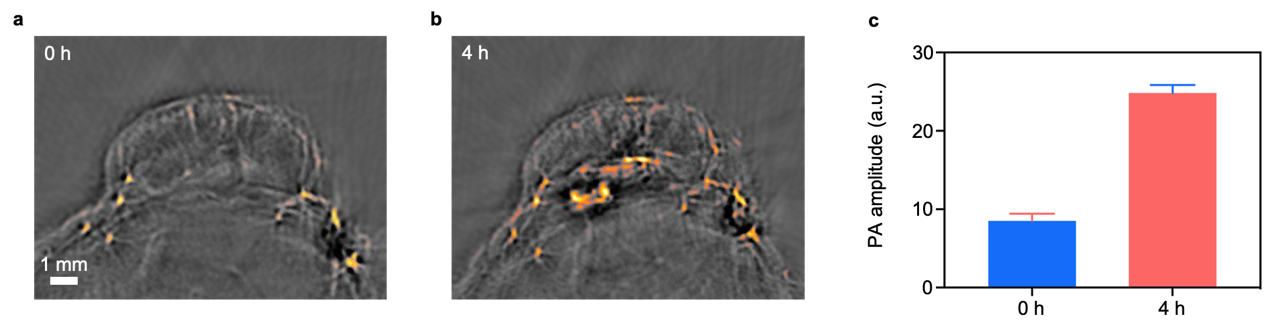


**Fig. S9** In vivo PACT images of tumor site by intratumorally treated with FTNPs (10 mg/kg, Fe_3_O_4_) at (a) 0 h without FTNPs injection and (b) 4 h post-injection. (c) Quantitative analysis of (a) and (b).


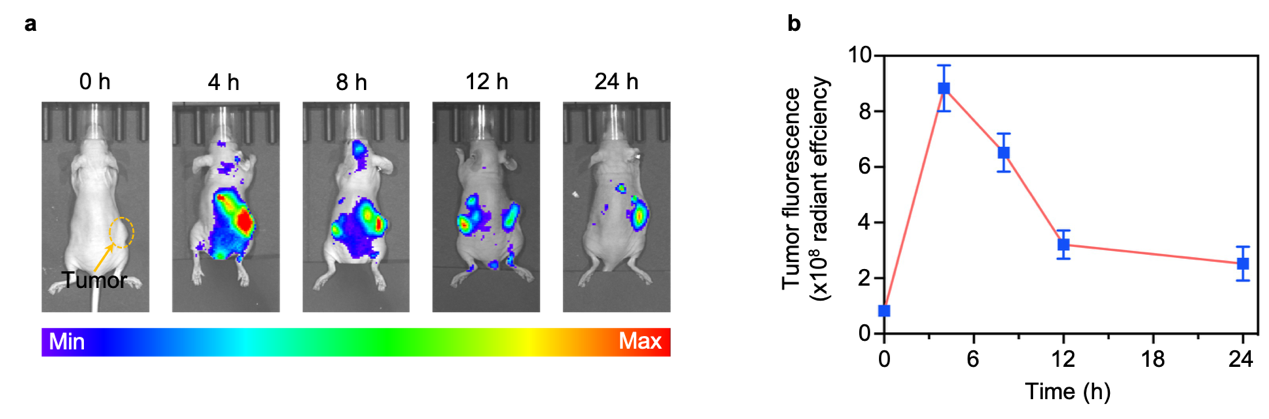


**Fig. S10** In vivo FL imaging (a) and quantified results (b) of mice after systemic administration of Cy5.5-labeled-FTNPs (10 mg/kg, Fe_3_O_4_).


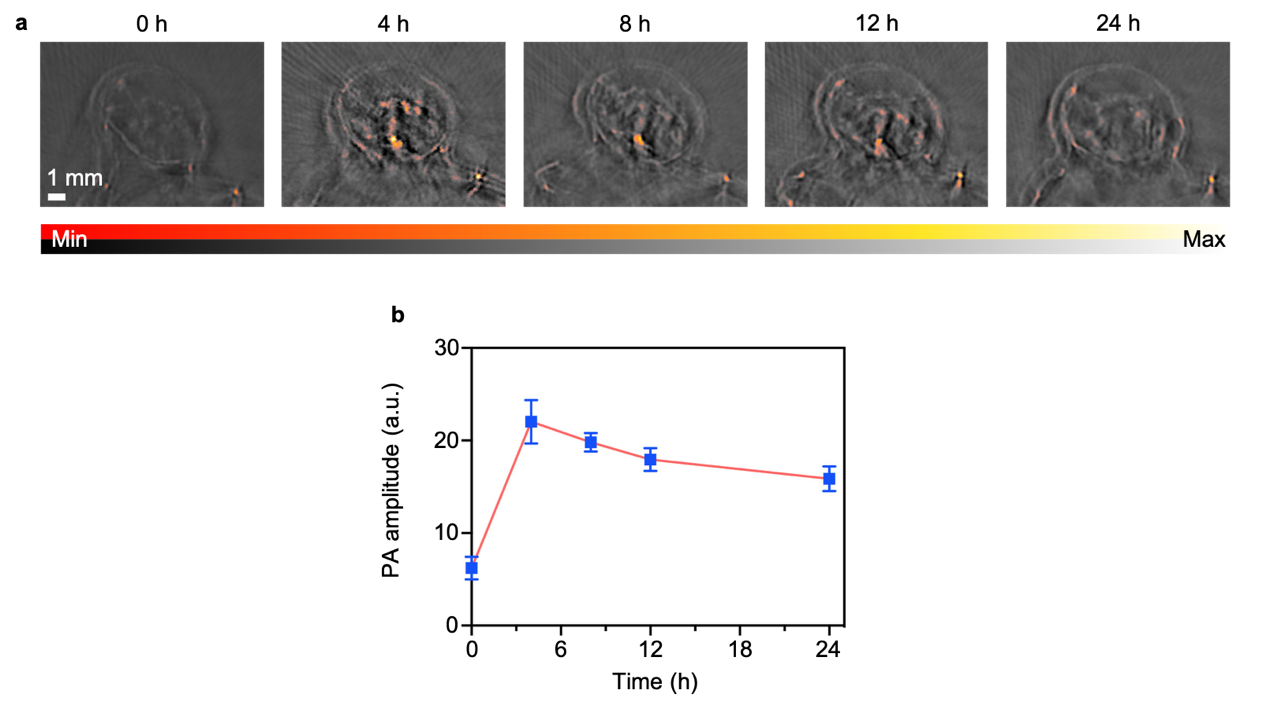


**Fig. S11** In vivo PACT imaging (a) and quantified results (b) of mice after systemic administration of FTNPs (10 mg/kg, Fe_3_O_4_).

**Fig. S12** The temperature of the tumor site and the temperature around the normal tissue after irradiation (808 nm, 2 W/cm^2^, 10 min).


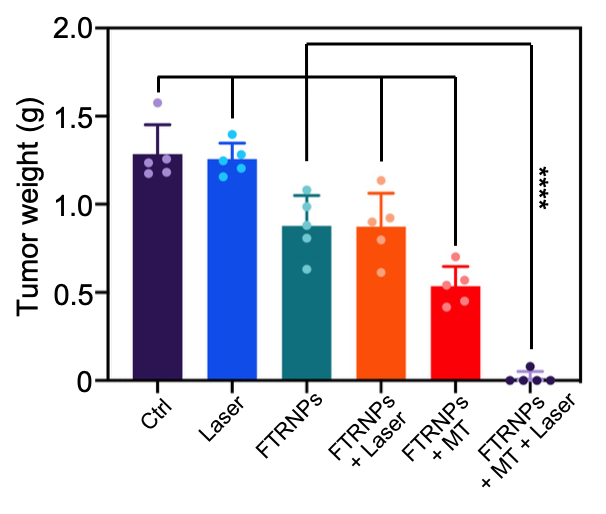


**Fig. S13** Tumor weights of mice after different treatments (PBS/FTRNPs (10 mg/kg, Fe_3_O_4_), with/without irradiation (808 nm, 2 W/cm^2^, 10 min), with/without magnetic targeting).
